# Resting Heart Rate Association with Cardiovascular Morbidity and Mortality and All-Cause Mortality According to Cluster of Cardiovascular Risk Factors—A Prospective Cohort Study with More than 20 Years of Follow-Up

**DOI:** 10.3390/jcm15187333

**Published:** 2026-09-21

**Authors:** Julio A. Carbayo-Herencia, Luis Miguel Artigao Ródenas, Marta Simarro-Rueda, Francisca Molina-Escribano, Francisca Navarro-Blazquez, Vicente Gil-Guillén, José R. Banegas, Juan Antonio Divisón Garrote

**Affiliations:** 1 Department of Clinical Medicine, Universidad Miguel Hernández Campus de San Juan, San Juan, 03550 Alicante, Spain; julio.carbayo.herencia@gmail.com (J.A.C.-H.); vte.gil@gmail.com (V.G.-G.); 2 GEVA (Grupo Enfermedades Vasculares Albacete), Chinchilla de Monte-Aragón, 02520 Albacete, Spain; lartigao02@gmail.com (L.M.A.R.); martasimr@gmail.com (M.S.-R.); paqui.molina@gmail.com (F.M.-E.); fnavarroblazquez@gmail.com (F.N.-B.); jadivison@telefonica.net (J.A.D.G.); 3 Zona 4 Primary Care Center, 02006 Albacete, Spain; 4 Chinchilla de Monte-Aragón Primary Care Center, Chinchilla de Monte-Aragón, 02520 Albacete, Spain; 5 Casasimarro Primary Care Center, Casasimarro, 16239 Albacete, Spain; 6 Unidad de Investigación HGUA Doctor Balmis, 03010 Alicante, Spain; 7 Department of Preventive Medicine and Public Health, Universidad Autónoma de Madrid and CIBERESP, 28029 Madrid, Spain; 8 Department of Medicine, Universidad Católica de Murcia (UCAM), 30107 Murcia, Spain

**Keywords:** heart rate, cardiovascular disease, mortality, cardiovascular risk, cohort study

## Abstract

**Objectives**: This study evaluated the association of resting heart rate (RHR) with major adverse cardiovascular events (MACEs) and all-cause mortality among adults without prior cardiovascular disease, stratified by baseline burden of cardiovascular risk factors (CVRFs). **Methods**: This was a prospective cohort study in a random sample of the general population aged ≥18 years in southeastern Spain. RHR was measured using an electrocardiogram. CVRFs (sedentary or light-to-moderate physical activity, body-mass index ≥ 25 kg/m^2^, non-HDL cholesterol ≥ 130 mg/dL, diabetes, hypertension, and smoking) were grouped in three clusters based on whether each subject has 0–2, 3, or ≥4 factors. During a mean 21.5-year follow-up, MACEs were collected and classified as all-cause mortality (outcome-1, *n* = 193 events) and combined cardiovascular morbidity and mortality (outcome-2, *n* = 291 events). Cox regression models were adjusted for multiple confounders. **Results**: The mean age of the participants (*n* = 1169) was 47.4 years (55.8% women). For every 10 bpm increase in RHR, the fully adjusted hazard ratio was 1.15 (95% CI: 1.03–1.28) for outcome-1 and 1.09 (1.00–1.20) for outcome-2. In the cluster with three CVRFs, the fully adjusted hazard ratios per 10 bpm RHR increase were 1.18 (95% CI 1.04–1.36) for outcome-1 and 1.15 (1.03–1.27) for outcome-2. In the clusters with 0–2 and ≥4 CVRFs, there were no statistically significant differences. **Conclusions**: RHR was independently associated with both cardiovascular events and all-cause mortality in the 3 CVRF cluster (intermediate cluster in terms of the burden of CVRFs), whose prognostic value may be clinically meaningful. These results highlight the importance of RHR and the comprehensive assessment of CVRFs in the primary prevention setting.

## 1. Introduction

Resting heart rate (RHR) is a simple variable that has long been studied; it influences life expectancy [1] and can predict cardiovascular (CV) and all-cause mortality [2]. It is measured directly (by pulse), via electrocardiogram (ECG), when measuring blood pressure (BP) with automatic out-of-office BP devices, and/or through ambulatory BP monitoring [3]. Agreement among the different methods is good [4]. RHR exhibits a circadian rhythm [5] and varies with body position [6]. There is still debate regarding normal values [7,8] and thresholds [9], and it has been suggested that its measurement should be standardized [10].

Numerous studies show an association between RHR and CV morbidity and mortality and all-cause mortality [11,12,13,14,15]. This association is more pronounced in low-risk, middle-aged men [16] and less so in older adults [17]. There is a relationship between RHR and mortality, but with differences based on age and sex [18,19]. Results from Regicor [20] and other studies in the general Japanese population predict long-term mortality [21]. Many studies link RHR and its variability to CV disease, CV mortality, and all-cause mortality, especially when increased RHR and high BP occur together. This association weakens when adjusted for other CV risk factors (CVRFs), and it is questionable whether reducing heart rate, whether through lifestyle changes or medication, is beneficial [22,23,24,25,26,27,28,29,30,31,32]. Palatini [3] and Zhang [2] emphasize this point and the importance of variability—a finding confirmed in older adults [33] and regarding their frailty [34]—and note that long-term variability in RHR and pulse pressure also increases the risk of all-cause mortality [35]. Other similar studies confirm the significance of this variability [36,37,38,39,40,41,42,43].

RHR is associated with exercise capacity and the risk of death in veterans [44]. In young adults, the risk of high RHR was slightly overestimated when prior physical fitness was not taken into account [45]. Exceeding the minimum level recommended by physical activity guidelines may reduce the risk of CV disease-related mortality associated with RHR [46]. It is clear that a high RHR is associated with the classic risk factors, but even after adjusting for all of them, it continues to predict mortality [47]; this relationship appears linearly starting at 60 bpm, but depends on underlying physical or pathophysiological status [45]. Mancia G. et al. [48]. and Grassi et al. [49] summarize the pathophysiology of increased RHR due to heightened sympathetic activity, which ultimately makes heart rate an independent CVRF in hypertensive patients.

Maintaining good CV health, which includes assessment of smoking status, physical activity, diet, body mass index (BMI), blood pressure, cholesterol, and glucose, reduces the likelihood of high RHR, regardless of sex, age, or ethnicity [50].

In virtually all established CV disease conditions and in chronic kidney disease (CKD), studies have linked high RHR to worse prognosis. In patients with a history of ischemic stroke, higher RHR has predictive value for major adverse CV events (MACEs) [51]. High RHR, along with other risk factors, is associated with fatal coronary events [52]. In patients with heart failure (ejection fraction < 40%), a higher heart rate was associated with a higher risk of death or transplantation after adjusting for age and sex [53]. A higher heart rate was associated with higher all-cause mortality and more CV events in patients with CKD not on dialysis [54]. Even in patients with chronic obstructive pulmonary disease (COPD), an increase in time-adjusted heart rate is associated with higher mortality, independent of other predictors, and is greater than the baseline heart rate [55].

Although attempts to pharmacologically reduce RHR appear to have less evidence-based support (perhaps with the exception of hypertensive patients with a heart rate > 80 beats per minute (bpm), as already included in clinical practice guidelines) [56], it does seem prudent to try to correct the cause of sympathetic–vagal imbalance through lifestyle changes such as reducing tobacco and alcohol consumption and increasing physical activity, which lower RHR and improve stress factors and insulin resistance; overall, current findings suggest that lowering RHR could be a potential target for reducing the risk of CV disease and premature mortality [56].

We examined a randomly selected, stratified cohort of the general population that was free from baseline CV disease and acute intercurrent conditions. Any acute intercurrent conditions that arose subsequently were recorded for the following weeks. The cohort had a low incidence of chronic conditions and limited use of medications capable of altering RHR. We therefore assessed whether RHR, measured by ECG, was independently associated with the onset of CV morbidity and mortality and all-cause mortality over more than 20 years of follow-up. Specifically, its prognostic association was assessed according to clusters of participants using three risk-factor groups (0–2, 3, or ≥4 CVRFs).

## 2. Material and Methods

### 2.1. Study Design and Participants

We carried out a prospective cohort study in a sample of the general population aged ≥18 years in the province of Albacete (southeast Spain), selected randomly and stratified in two stages. Initially, 1322 subjects participated and were assessed between November 1992 and June 1994; a second examination was conducted in 2004–2006, a third in 2014–2016, and a fourth in 2019. Briefly, the cohort was recruited through a 3-stage sampling design of all the inhabitants in the province of Albacete registered in the 1991 census. The first phase consisted of a stratified sample based on the population of the locality in which the participants resided (capital, 39.7%; >10,000, 23.3%; 2001–10,000, 20.8%; 501–2000, 14.5%, and <501, 1.7%); the second phase was based on a cluster sampling of the municipalities contained in these groups, and the final phase was a simple random sampling. The sample size in each municipality was proportional to the size of its population. In this study, 153 individuals were excluded due to previous cardiovascular disease, type-1 diabetes, insufficient weight, or lack of clinical analyses, leaving a final sample of 1169 participants. The methodology was described previously [57,58].

### 2.2. Heart Rate Measurements and Other Study Variables

The mean value of RHR measured at rest with an ECG was considered. Hypertension was defined as systolic BP (SBP) ≥ 140 mmHg or diastolic BP (DBP) ≥ 90 mmHg or being on antihypertensive drug treatment. The average of two consecutive BP measurements was used, taken with calibrated mercury sphygmomanometers, with cuffs appropriate for the size of the arm, in a seated position and under standard conditions, with prior training and certification of the research physicians. Diabetes mellitus (DM) was considered if the baseline blood glucose was ≥126 mg/dL, confirmed on two occasions, or if the subject was undergoing treatment; hypercholesterolemia was considered if the total cholesterol was ≥200 mg/dL or if the subject was undergoing treatment and exhibited elevated non-high-density lipoprotein (HDL) cholesterol (total cholesterol minus non-HDL cholesterol) if ≥130 mg/dL. BMI was obtained by dividing weight in kg by height in meters squared (kg/m^2^), defining obesity as BMI ≥ 30 kg/m^2^ and overweight ≥ 25 kg/m^2^. Smoking was defined as reporting tobacco use at the baseline visit, regardless of the amount or duration. Lastly, physical activity was classified as mild, moderate, or severe, depending on work activity.

### 2.3. Cluster Cardiovascular Risk Factors Assessment

The sample was divided into three groups according to whether each study subject had 0–2, 3, or ≥4 CVRFs among the following predefined factors: 1 sedentary or light-to-moderate physical activity; 2 Overweight or obesity (≥25 kg/m^2^); 3 non-HDL cholesterol ≥ 130 mg/dL, 4 DM (yes/no); 5 hypertension (yes/no); and 6 smoking (yes/no). In other words, the four classic CVRFs, in addition to physical activity and weight [59].

### 2.4. Follow-Up and Events Occurred

Participants were monitored and followed for an average of 21.5 ± 6.9 years to assess MACEs, which were defined as hospitalization for heart failure, acute coronary syndrome, angina of any type, acute myocardial infarction, cardiac revascularization, transient ischemic attack, stroke, severe peripheral ischemic artery disease, or death from cardiovascular causes. Cardiovascular mortality included causes in which the death certificate specified a MACE as the underlying cause of death. All-cause mortality was recorded. The criterion for defining each MACE was that the diagnosis of the episode was clearly documented in the patients’ electronic hospital and/or primary-care medical records. In the assessment of MACEs, the first episode that occurred during follow-up was considered, noting the diagnosis and date of the event. For the purposes of the analysis, events were classified as all-cause mortality (outcome-1) or combined cardiovascular morbidity and mortality (outcome-2).

### 2.5. Statistical Analysis

Qualitative variables are presented as absolute numbers and percentages, and quantitative variables as means and standard deviations. To compare means between variables with two categories, Student’s *t*-test for independent groups was used, and chi-square was used for qualitative variables.

To compare more than two means with categories ordered by the number of cardiovascular risk factors (0–2, 3, or ≥4 risk factors), the Jonckheere–Terpstra test was used. The relationship between the ordered cardiovascular risk and the remaining qualitative variables was assessed using the Mantel–Haenszel linear-trend test.

RHR was categorized into two groups in each of the dependent variables (outcome-1 and outcome-2), applying in its calculation the median value for both the sample as a whole and each of the subgroups defined according to the number of cardiovascular risk factors, as described above. The probability of survival for these groups was calculated using the Kaplan–Meier test, comparing them using the logarithmic rank test.

Cox regression models were used to examine the prognostic significance of RHR, after checking the proportionality of instantaneous risks graphically and using Schoenfeld residuals [60]. The models were built for the total sample and for each category defined by the number of cardiovascular risk factors. The hazard ratio results are shown for each 10 bpm increase in heart rate; when heart rate was assessed as a dichotomous variable, the lower cutoff point was taken as reference.

The variables included in the adjustment were those considered clinically relevant for the associations studied and that could confound the results: age, sex, hypertension, type-2 diabetes, BMI, smoking status, non-HDL cholesterol, and triglyceride/HDL cholesterol ratio. Incidence rates per 10,000 persons per year were calculated based on the years of follow-up for each category defined by qualitative RHR and cardiovascular risk factors.

Given that the average RHR was different in women than in men, the term sex x RHR interaction was introduced in the models for each of the CVRF groups and in the sample with all variables incorporated. The interaction term did not reach statistical significance, so the results are presented without it.

A *p*-value < 0.05 was considered significant. Statistical analysis was performed using SPSS Statistics for Windows, Version 26.0, IBM Corp. (Armonk, NY, USA).

### 2.6. Ethical Issues

This study was conducted in accordance with international guidelines on clinical research (the World Medical Association’s Declaration of Helsinki). All patients signed an informed consent form, and the study was approved by the Clinical Research Ethics Committee of Albacete University Hospital (GEVA study, registry #7446, Junta de Comunidades de Castilla-La Mancha, Spain). The presentation of this study follows the STROBE initiative statement (Strengthening the Reporting of Observational Studies in Epidemiology) applied to cohort studies [60].

## 3. Results

### 3.1. Participant Characteristics

**Figure 1:**
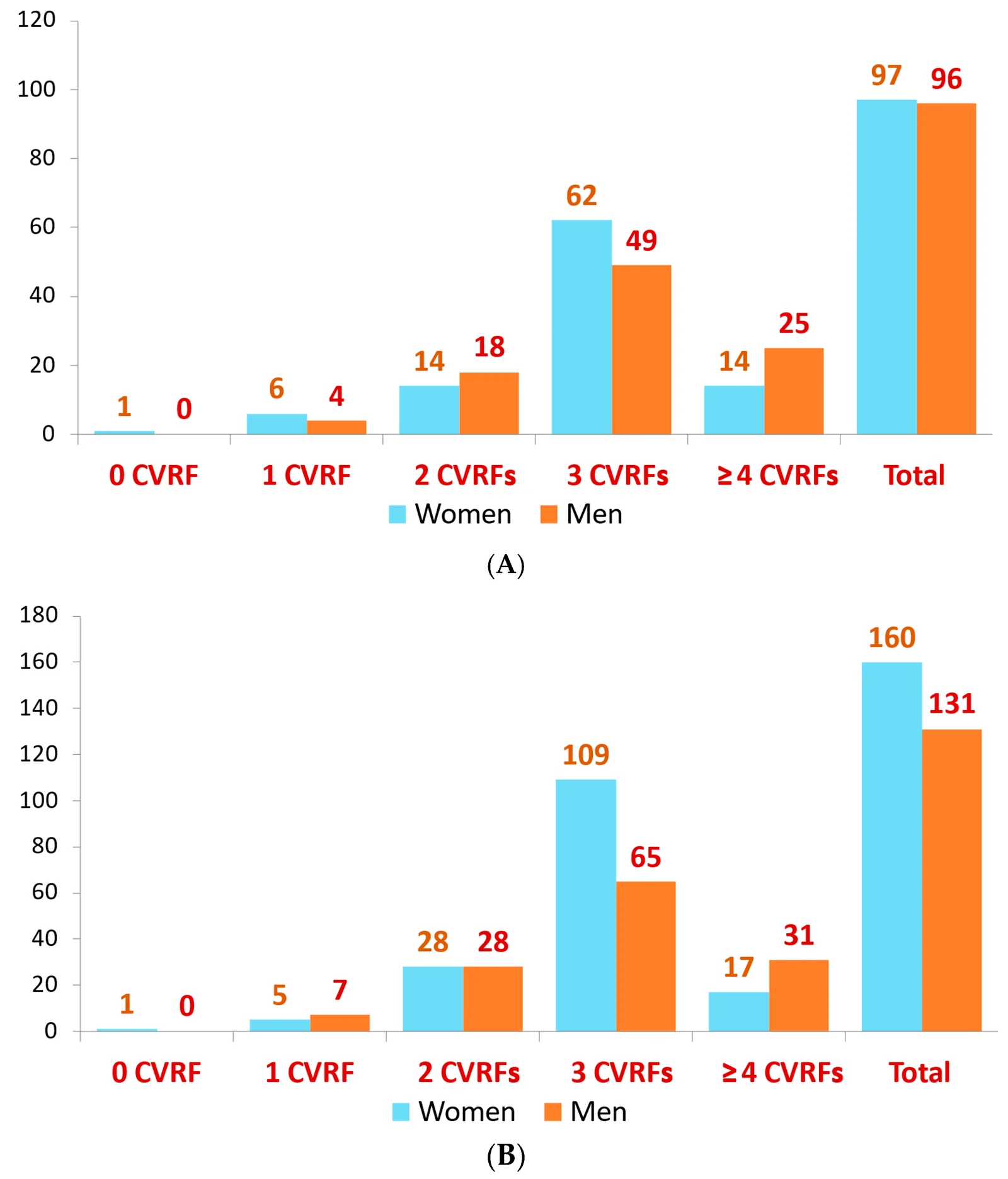
(**A**) Deaths related to the number of CVRFs, by sex. (**B**) Number of events due to cardiovascular morbidity and mortality, by sex.

Of the 1169 participants, 652 (55.8%) were women and 517 (44.2%) were men. The mean baseline age of the 1169 participants was 47.4 ± 17.4 years (55.8% women). The mean follow-up time was 21.5 ± 6.9 years (median, 25.5 years). The mean HR was 73.4 ± 12.6 bpm (75.6 ± 12.8 in women and 70.8 ± 11.8 bpm in men; *p* < 0.001). For greater clarity, the events observed in the cardiovascular risk factor subgroups of the patients, by sex, are shown in Figure 1. The number of deaths and cardiovascular events increased with the number of risk factors up to 3; however, an exception was observed in the group with ≥4 risk factors, whereas among those with 3 risk factors, the number of deaths and cardiovascular episodes was higher in women.

Table 1 shows the results considering dichotomized RHR; the cutoff point is the median value of the sample. In general, an increase in the values of the study variables and outcomes was observed in highest category of the median cutoff point.

**Figure 2:**
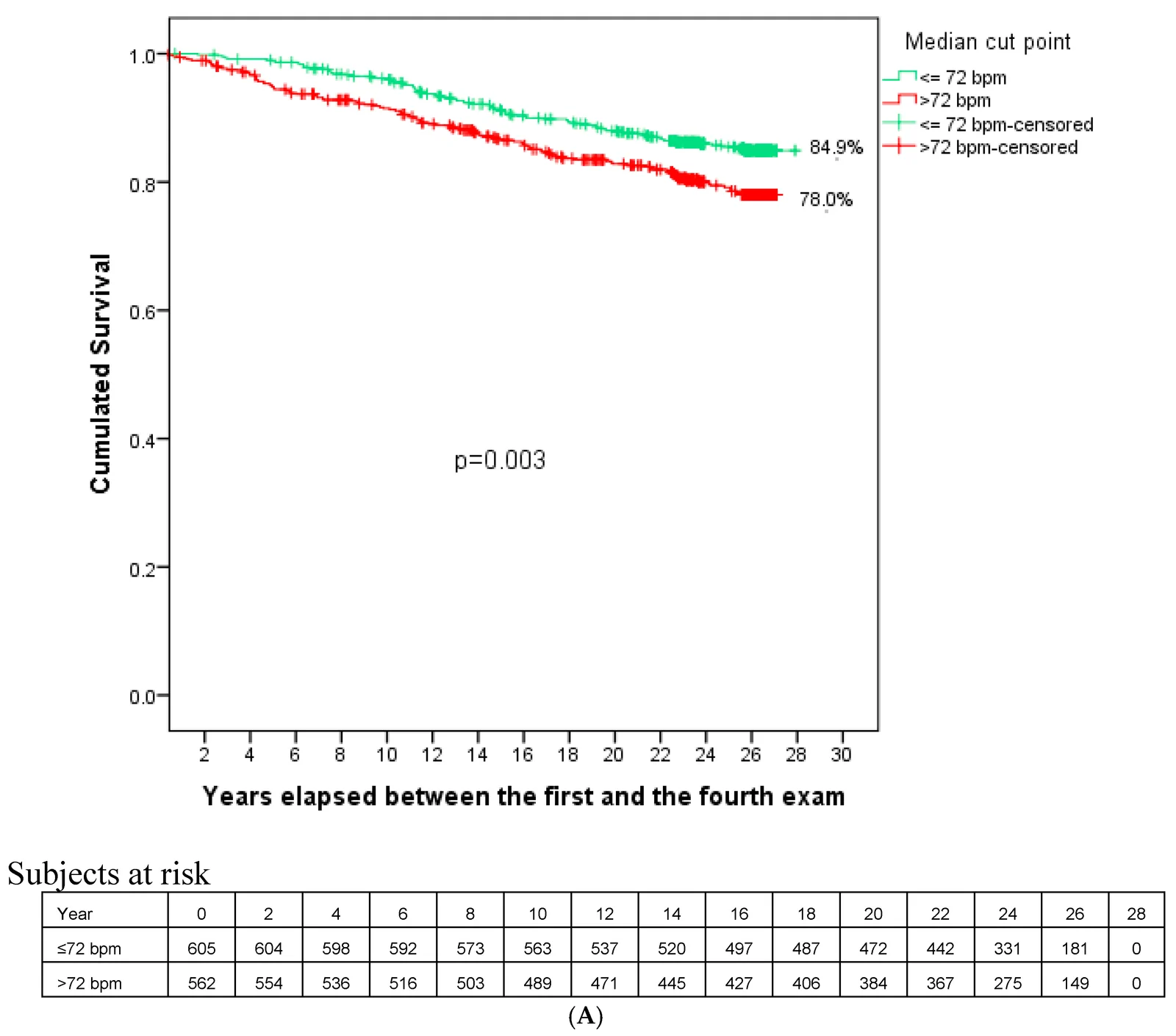

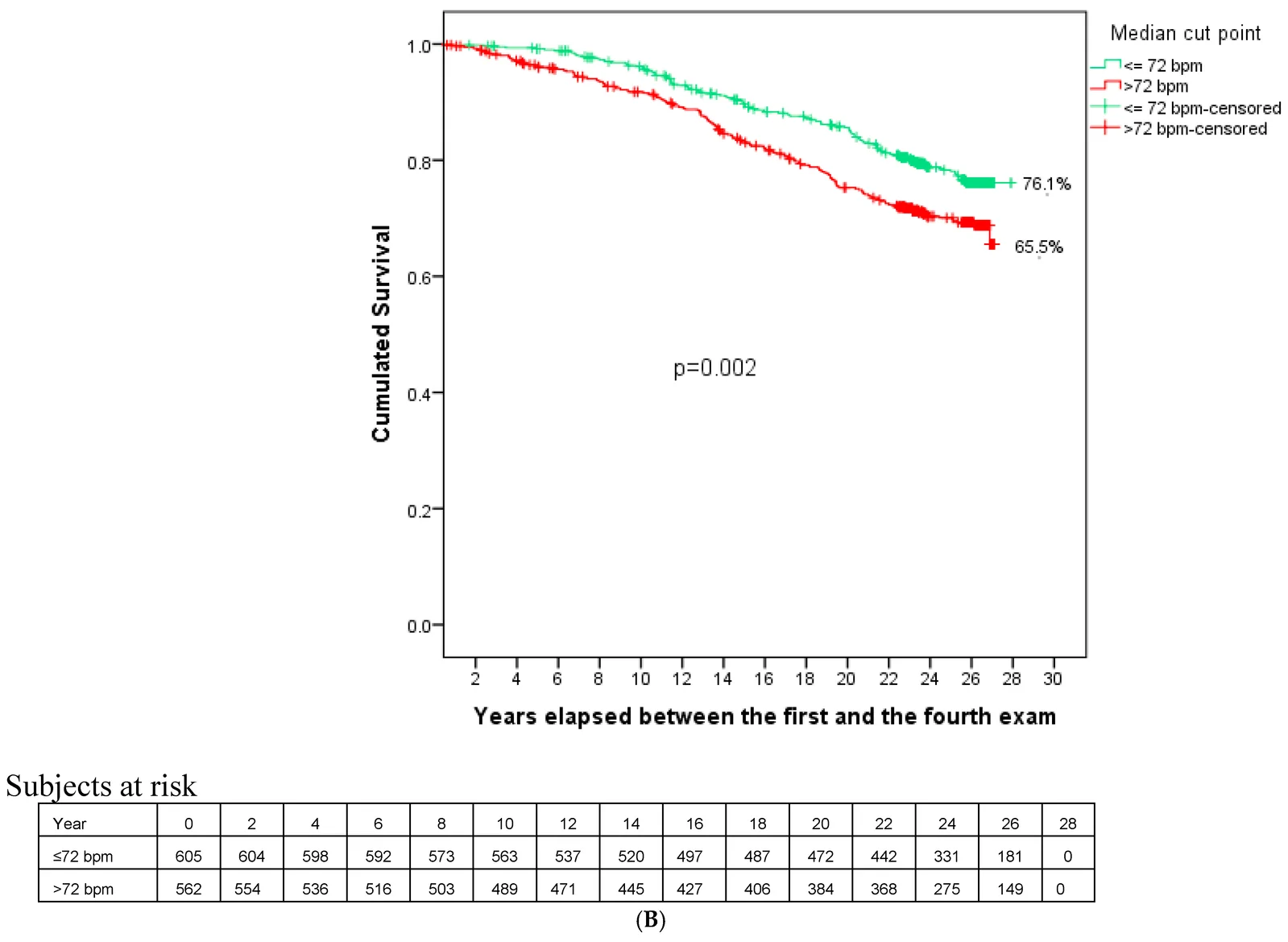
Survival curves for all-cause mortality (**A**) and the combination cardiovascular morbidity and mortality (**B**).

During follow-up, 193 people (16.5%) died (outcome-1) and 291 (24.9%) presented outcome-2. Figure 2 shows a significantly more pronounced downward trend in cumulative survival for both outcomes in the groups with higher RHR.

Both age and some of the values of the other variables tended to increase as the number of risk factors increased from 0–2 to 3, with much higher sample size than the group with ≥4 CVRFs (Table 2). While mean age and the frequency of male gender, tobacco smoking, diabetes, overweight, total cholesterol, and non-HDL cholesterol were higher in the 4 CVRF cluster, the frequency of hypertension, mean BMI, obesity, triglycerides, and mean TG/non-HDL ratio were higher in the 3+ CVRF cluster.

The following events occurred for outcome-1: 43 deaths in the 0–2 risk factors group; 111 when subjects had 3 risk factors, and 39 when they had ≥4. In outcome-2, there were 69 cardiovascular events in the 0–2 CVRF group; 174 in the 3 CVRF group, and 48 in the ≥4 CVRF group (Table 3). The highest incidence of episodes (outcome-1 and outcome-2) was observed in persons with 3 and ≥4 clusters of CVRFs, as well as in those with a higher heart rate (Table 3).

The years of follow-up according to the heart rate category were 13.437 for ≤72 bpm and 11.655 for >72. In each CVRF accumulation category, the figures were: 0–2 CVRFs: 12.612; 3 CVRFs: 9.795; 4 or more CVRFs: 2.685, and 25.092 in the entire sample. The incidence rate is expressed per 10.000 person/years.

### 3.2. Association Between Resting Heart Rate and Outcomes

In outcome-1, for every 10 bpm increase in RHR, the fully adjusted hazard ratio was 1.15 (95% CI: 1.03–1.28); in outcome-2, it was 1.09 (1.000–1.20) (Table 4). Regarding the age- and sex-adjusted model, a heart rate exceeding 72 bpm presents a hazard ratio of 1.53 (1.15–2.05) in outcome-1 and 1.43 (1.13–1.81) in outcome-2, based on HR ≤ 72 bpm as the reference in both cases.

Only in the 3 CVRFs group were the differences statistically significant for both outcomes, whether assessed by the 10 bpm increase or the median HR of each group: per 10 bpm increase in RHR the fully adjusted hazard ratios were 1.18 (95% CI 1.04–1.36) for outcome-1 and 1.15 (1.03–1.27) for outcome-2 (Table 5).

## 4. Discussion

This study, conducted in a middle-aged general population in a primary-prevention setting, shows a prognostic value (>20 years) of different heart rate values in the overall sample. The description of baseline characteristics shows that subjects in the upper half of the RHR median have significantly elevated levels of classic risk factors (with the exception of cholesterol), as reported in the literature for diabetes mellitus [61], systolic and diastolic blood pressure and hypertension [62], and BMI and obesity [63].

In our study, the separation of survival curves in both outcomes can be seen from the first year of follow-up. In fact, after two decades of follow-up of the Framingham population, with RHR measured by ECG, the authors concluded that higher RHR increased the risk of CV disease, particularly heart failure, and all-cause mortality. They proposed that these adverse outcomes may have a multifactorial origin, including proatherogenic effects related to increased shear stress and the progression of atherosclerosis, adverse effects on myocardial energy (explaining the increase in heart failure), autonomic dysfunction, and poorer physical fitness.

Changes in both outcomes examined were observed for every 10 bpm increase in RHR, specifically a 15% increase in risk for outcome-1 and 9% (very close to statistical significance) increase for outcome-2. These data are consistent with other studies that found that for every 10 bpm increase in heart rate, all-cause mortality increased by 9% and cardiovascular mortality by 8% [2,14]. Significant changes above the median RHR were also observed in both outcomes after full adjustment (outcome-1: 45% increase in risk, and outcome-2: 36%). It therefore appears that managing heart rate could be a goal in managing the cardiovascular continuum, either with lifestyle changes or with drugs for primary or secondary prevention [64]. Böhm et al. assessed RHR and cardiovascular outcomes in diabetic and non-diabetic patients with high cardiovascular risk, suggesting that above 75–80 bpm, reducing heart rate should be considered based on its predictive ability [32]. These same authors had previously reviewed RHR as an indicator and emerging risk factor for cardiovascular disease [65]. Also, we know that high RHR is related to classic risk factors, perhaps with the exception of age—in the very elderly there may be no association between resting RHR and mortality [17].

Finally, when we examined the association of RHR, as measured by ECG, with outcome-1 and outcome-2 based on the burden or number of risk factors in the evaluated groups, we found a clear and significant increase in the risk in the group with 3 CVRFs: 18.4% for every 10 bpm in outcome-1 and 14.9% in outcome-2. Similarly, in the same group (3 CVRFs), when the study subjects were above the median for their group (73 bpm), the increase in outcome-1 and outcome-2 was 70.5% and 59.0%, respectively. This increase was not observed in subjects with fewer risk factors (0–2) and was not statistically significant in the group with the highest cumulative number of risk factors, presumably due to the small sample size in this subgroup and to potentially greater control and compliance. Also, clinically, the 3 CVRF group was characterized by a higher prevalence of hypertension, obesity, hypertriglyceridemia, and an elevated TG/non-HDL ratio—a profile strongly linked to metabolic syndrome and hyperactivity of the sympathetic nervous system. We suggest that, in this specific cardiometabolic substrate, an RHR may act as an amplifier of CV risk. Conversely, in the group with ≥4 CVRFs, characterized by a higher burden of traditional atherogenic factors (smoking, diabetes, advanced age, non-HDL cholesterol), the prognostic value of these classic factors may saturate the model, diluting the independent impact of RHR.

Some authors [48] have emphasized that RHR is significantly associated with organ damage and morbidity and mortality in a wide range of patients, including those with hypertension, for whom guidelines recommend ECG testing to detect left ventricular hypertrophy [56] This may mean that having a reliable RHR measurement could serve as a basis for examining “variability” 43, with the subsequent and numerous blood pressure and RHR measurements that are routinely performed (in the clinic and at home) adding value to the isolated RHR. Other authors summarize the pathophysiology of increased heart rate due to increased sympathetic activity and even claim that this is an independent cardiovascular risk factor in hypertensive patients [49].

Olshansky et al. found that RHR predicts total and cardiovascular mortality, being especially relevant if there is underlying disease (inverse causality), reflecting in that case its severity or, conversely, reflecting good general health in “non-diseased” individuals [66]. This would reflect the general health of our population, since people with cardiovascular disease were excluded beforehand (and obviously their initial examination was postponed when they presented acute conditions). These authors also concluded that therapies that only reduce heart rate are not necessarily useful and may not improve outcomes, while changes that improve the underlying cause (whether in disease or “health”) and thus decrease heart rate would improve those outcomes [66]. Finally, the authors initially sought to determine whether RHR had an independent predictive association with cardiovascular risk as calculated using predictive models. However, this analysis was not possible because the age inclusion criteria of the classical models substantially reduced the available sample size, and RHR is itself included as a in these models. A recent study reports an attempt to apply this approach [17].

### 4.1. Strengths and Limitations

It should be noted that the study sample was randomly selected from the general population (not receiving care at health centers) in a province in southeastern Spain, which facilitates the generalization of the results, although the relatively small sample size partly weakens its value. Precisely the relatively small sample size of this study made it difficult to analyze men and women separately, which could contribute to a better understanding of RHR as a risk factor.

Other studies have measured RHR using methods other than ECG and have reported similar results; however, some obtained measurements on various occasions, allowing them to evaluate RHR “variability” [48], which could not be assessed in our study.

We attempted to minimize information bias, but when using diverse records (especially primary care and hospital medical records with varying quality), difficulties always arise. However, each participant’s medical records were thoroughly reviewed by two or more researchers from the group when any diagnostic doubts arose regarding MACEs or the cause of death. In the analysis of mortality, on very rare occasions it was necessary to resort to death certificates or direct information from family members.

Another limitation is not using a prediction model such as SCORE and SCORE-OP (which are accepted in our setting) or one of the classic American models because the equations are valid only when derived from populations with similar risk factors and comparable cardiovascular morbidity and mortality. If this is not the case, adjustments are necessary that do not always yield acceptable predictions. Note that it is impossible to compare genetic backgrounds, environmental factors, lifestyles, and levels of medical care across different populations, even if they have similar risk factors and rates of cardiovascular disease. Since we cannot use our own model (heart rate is included in it and would cause overfitting), we decided to use three groups with “accumulated” CVRFs.

It was noted that some participants were being treated with medications (beta-blockers, non-dihydropyridine calcium channel blockers, or digoxin) that lower heart rate; however, this applied to only 40 (3.4%) participants, and there were no significant differences between them and the rest of the sample (73.4 ± 12.6 vs. 74.9 ± 14.0). While noting that this is a randomly selected cohort, the number of dropouts could call the study into question; in our case, it represents 16.3%, which is acceptable for a general population.

On the other hand, RHR measurements obtained at distant time points were evaluated in relation to cardiovascular events and deaths recorded as endpoints. Possible changes in CVRFs during the long follow-up period and treatments that could modify heart rate were not taken into account. Similar limitations occurred in early cohort studies that evaluated adverse cardiovascular events and deaths during follow-up.

### 4.2. Conclusions and Perspectives

In conclusion, RHR is an independent risk factor for CV disease and all-cause mortality in the general population in the context of primary prevention. However, its prognostic value depends on the patient’s risk profile, with its clearest value being observed in individuals with a predominantly cardiometabolic risk profile (3 CVFRs), characterized by hypertension, obesity, and hypertriglyceridemia.

While awaiting new studies that provide data on the predictive ability of RHR based on CV risk calculated by various models, which, incidentally, have always been questioned, this study demonstrates its prognostic value in this specific general population with an average number of risk factors (3 out of 6: 4 classic risk factors plus physical activity and weight). This group accounts for nearly half of the study population, and its prospective association with MACEs and all-cause mortality is significant and of particular clinical interest.

## Figures and Tables

**Table 1:** Baseline characteristics of the participants according to their resting heart rate above or below median.

	Total (*n* = 1169)	HR ≤ 72 bpm (*n* = 606; 51.8%)	HR > 72 bpm (*n* = 563; 48.2%)	*p*
Sex, *n* (%) Female Male	652 (55.8) 517 (44.2)	295 (48.7) 311 (51.3)	357 (63.4) 206 (36.6)	<0.001
Age (yrs)	47.4 (17.4)	46.1 (17.1)	48.8 (17.7)	0.008
Heart rate (bpm)	73.4 (12.6)	64.0 (5.9)	83.6 (9,8)	<0.001
Smoking, *n* (%)	376 (32.2)	200 (33.0)	176 (31.3)	0.524
SBP (mmHg)	132.5 (21.7)	130.1 (20.8)	135.2 (22.4)	<0.001
DBP (mmHg)	81.5 (12.4)	80.1 (12.0)	83.1 (12.6)	<0.001
Hypertension, *n* (%)	499 (42.7)	223 (36.8)	276 (49.0)	<0.001
Type-2 diabetes, *n* (%)	110 (9.4)	44 (7.3)	66 (11.7)	0.009
BMI, kg/m^2^	27.5 (4.8)	26.9 (4.3)	28.2 (5.1)	<0.001
Plasma glucose (mg/dL)	100.1 (28.0)	97.1 (23.5)	103.4 (31.8)	<0.001
Total cholesterol (mg/dL)	199.6 (39.0)	197.8 (37.0)	201.4 (40.9)	0.116
Non-HDL cholesterol (mg/dL)	152.7 (39.3)	150.9 (37.6)	154.6 (41.1)	0.115
HDL cholesterol (mg/dL)	46.9 (11.9)	46.9 (11.7)	46.8 (12.3)	0.961
Triglycerides (mg/dL)	102.7 (70.2)	100.2 (71.0)	105.3 (69.2)	0.216
Triglycerides/HDL cholesterol ratio	2.5 (2.3)	2.5 (2.5)	2.5 (2.1)	0.654
BMI categories, *n* (%): Normal Overweight Obesity	387 (33.1) 468 (40.0) 314 (26.9)	224 (37.0) 260 (42.9) 122 (20.1)	163 (29.0) 208 (36.9) 192 (34.1)	<0.001

HR: heart rate; SBP: systolic blood pressure; DBP: diastolic blood pressure; BMI: body mass index; HDL: high-density lipoprotein. The HR value is the median value of the sample. Data are presented as mean (standard deviation) for quantitative variables and as exact number (percentage) for qualitative variables.

**Table 2:** Baseline characteristics of the participants according to cluster risk factors.

*n* = 1169	0–2 Risk Factor (*n* = 531; 45.4%)	3 Risk Factors (*n* = 497; 42.5%)	4 or More Risk Factors (*n* = 141; 12.1%)	*p*
Females (*n* = 652; 55.8%) Males (*n* = 517; 44.2%)	305 (57.4%) 226 (42.6%)	282 (56.7%) 215 (43.3%)	65 (46.1%) 76 (53.9%)	0.052
Age (yrs)	39.4 (15.6)	55.1 (15.0)	50.7 (19.0)	<0.001
Tobacco smokers *n* (%)	158 (29.8%)	125 (25.2%)	93 (66.0%)	<0.001
SBP (mmHg)	120.8 (15.0)	144.4 (21.2)	134.7 (22.0)	<0.001
DBP (mmHg)	75.6 (9.8)	87.8 (11.7)	81.6 (12.6)	<0.001
Heart rate (bpm)	71.4 (11.9)	74.9 (13.2)	76.0 (12.0)	<0.001
Hypertension, *n* (%)	57 (10.7%)	362 (72.8%)	80 (56.7%)	<0.001
Type-2 diabetes, *n* (%)	9 (1.7%)	77 (15.5%)	24 (17.0%)	<0.001
BMI (kg/m^2^)	24.9 (3.5)	30.1 (4.6)	27.9 (4.4)	<0.001
BMI categories, *n* (%)				
Normal	324 (61.0%)	31 (6.2%)	32 (22.7%)	
Overweight	152 (28.6%)	244 (49.1%)	72 (51.1%)	
Obesity	55 (10.4%)	222 (44.7%)	37 (26.2%)	<0.001
Plasma glucose (mg/dL)	93.3 (16.0)	106.04 (34.0)	104.7 (33.9)	<0.001
Total cholesterol (mg/dL)	184.2 (37.8)	219.1 (30.2)	188.4 (40.7)	<0.001
Non-HDL cholesterol (mg/dL)	134.6 (37.0)	174.2 (29.7)	144.7 (40.3)	<0.001
HDL cholesterol (mg/dL)	49.6 (12.1)	44.8 (11.4)	43.7 (11.2)	<0.001
Triglycerides (mg/dL)	82.9 (66.1)	122.6 (71.9)	106.7 (57.5)	<0.001
Triglycerides/HDL cholesterol ratio	1.89 (1.85)	3.11 (2.63)	2.72 (2.16)	<0.001

SBP: systolic blood pressure; DBP: diastolic blood pressure; BMI: body mass index; HDL: high-density lipoprotein; bpm: beats per minute. Data are presented as mean (standard deviation) for quantitative variables and as exact number (percentage) for qualitative variables.

**Table 3:** Incidence rates of all-cause mortality and the combined cardiovascular morbidity and mortality according to heart rate category and number of CVRFs.

	All-Cause Mortality		CV Morbidity and Mortality	
	No. Events/Total	Incidence Rate (95% CI)	No. Events/ Total	Incidence Rate (95% CI)
Heart rate				
≤72 bpm (*n* = 606)	83/606	62 (48–75)	130 (606)	97 (80–113)
>72 bpm (*n* = 563)	110/563	94 (77–112)	161 (563)	138 (117–159)
Number of CVRFs				
0–2 (*n* = 531)	43/531	34 (24–44)	69/531	55 (42–68)
3 (*n* = 497)	111/497	113 (92–134)	174/497	178 (151–204)
4 or more (*n* = 141)	39/141	145 (100–191)	48/141	179 (128–229)
Total (*n* = 1169)	193/1169	77 (66–88)	291/1169	116 (103–129)

CVRFs: cardiovascular risk factors; CV: cardiovascular; CI: confidence interval; bpm: beats per minute.

**Table 4:** Association of heart rate with all-cause mortality and with the combination of cardiovascular morbidity and cardiovascular mortality.

	Hazard Ratio (95% CI)					
All-cause mortality	Crude	*p*	Age and sex adjusted	*p*	Fully adjusted	*p*
Per 10 bpm increase in HR	1.20 (1.08–1.33)	<0.001	1.20 (1.07–1.32)	0.001	1.15 (1.03–1.28)	0.013
HR ≤ 72 bpm HR > 72 bpm	1 1.53 (1.15–2.04)	0.003	1 1.53 (1.15–2.05)	0.004	1 1.45 (1.09–1.94)	0.012
Cardiovascular morbidity plus Cardiovascular mortality	Crude	*p*	Age and sex adjusted	*p*	Fully adjusted	*p*
Per 10 bpm increase in HR	1.14 (1.04–1.24)	0.004	1.13 (1.03–1.23)	0.012	1.09 (1.00–1.20)	0.06
HR ≤ 72 bpm HR > 72 bpm	1 1.45 (1.15–1.82)	0.002	1 1.43 (1.13–1.81)	0.003	1 1.36 (1.08–1.72)	0.01

HR indicates heart rate. The variables included in the full adjustment were age, sex, heart rate (shown in this table, both quantitatively [every 10 beats/minute] and categorized into the two exposed groups), high blood pressure, type-2 diabetes, body mass index, smoking status, non-HDL cholesterol, and triglycerides/HDL cholesterol ratio.

**Table 5:** Association of heart rate with all-cause mortality and with the combination of cardiovascular morbidity and mortality, according to CVRF cluster.

	0–2 CVRFs (*n* = 531)		3 CVRFs (*n* = 497)		4 or more CVRFs (*n* = 141)	
	Hazard ratio (95% CI)	*p*	Hazard ratio (95% CI)	*p*	Hazard ratio (95% CI)	*p*
All-cause mortality						
Per 10 bpm increase in HR	1.03 (0.78–1.36)	0.813	1.184 (1.04–1.36)	0.011	1.03 (0.78–1.36)	0.846
Median HR *	0.86 (0.47–1.62)	0.658	1.705 (1.16–2.51)	0.007	1.60 (0.83–3.10)	0.165
Cardiovascular morbidity plus cardiovascular mortality						
Per 10 bpm increase in HR	1.04 (0.84–1.29)	0.699	1.149 (1.03–1.27)	0.014	0.84 (0.66–1.07)	0.159
Median HR *	1.23 (0.74–2.02)	0.426	1.590 (1.17–2.16)	0.003	0.70 (0.39–1.26)	0.740

HR: heart rate; bpm: beats per minute. CVRFs: cardiovascular risk factors. The variables included in the full adjustment were age, sex, high blood pressure, type-2 diabetes, body mass index, smoking status, non-HDL cholesterol, and triglycerides/HDL cholesterol ratio. * Median heart rate in each subgroup: 0–2 CVRFs: 70 bpm; 3 CVRFs: 73 bpm; ≥4: 76 bpm.

## Data Availability

The data that support the findings of this study are available from the corresponding author upon reasonable request.

## References

[1] Levine H.J. (1997). Rest Heart Rate and Life Expectancy. J. Am. Coll. Cardiol..

[2] Zhang D., Shen X., Qi X. (2016). Resting Heart Rate and All-Cause and Cardiovascular Mortality in the General Population: A Meta-Analysis. CMAJ.

[3] Palatini P., Rosei E.A., Casiglia E., Chalmers J., Ferrari R., Grassi G., Inoue T., Jelakovic B., Jensen M.T., Julius S. (2016). Management of the Hypertensive Patient with Elevated Heart Rate: Statement of the Second Consensus Conference Endorsed by the European Society of Hypertension. J. Hypertens..

[4] Sobieraj P., Leśniewski M., Sawicka A., Siński M., Lewandowski J. (2024). Agreement between Resting Heart Rate Measured by Unattended Automated Office and Office Blood Pressure Measurement, Ambulatory Blood Pressure Monitoring, or Electrocardiography. J. Clin. Hypertens..

[5] Nakagawa M., Iwao T., Ishida S., Yonemochi H., Fujino T., Saikawa T., Ito M. (1998). Circadian Rhythm of the Signal Averaged Electrocardiogram and Its Relation to Heart Rate Variability in Healthy Subjects. Heart.

[6] Kristal-Boneh E., Harari G., Weinstein Y., Green M.S. (1995). Factors Affecting Differences in Supine, Sitting, and Standing Heart Rate: The Israeli CORDIS Study. Aviat. Space Environ. Med..

[7] Spodick D.H., Raju P., Bishop R.L., Rifkin R.D. (1992). Operational Definition of Normal Sinus Heart Rate. Am. J. Cardiol..

[8] Spodick D.H. (1993). Redefinition of Normal Sinus Heart Rate. Chest.

[9] Palatini P. (1999). Need for a Revision of the Normal Limits of Resting Heart Rate. Hypertension.

[10] Vogel C.U., Wolpert C., Wehling M. (2004). How to Measure Heart Rate?. Eur. J. Clin. Pharmacol..

[11] Ho J.E., Larson M.G., Ghorbani A., Cheng S., Coglianese E.E., Vasan R.S., Wang T.J. (2014). Long-term Cardiovascular Risks Associated With an Elevated Heart Rate: The Framingham Heart Study. J. Am. Heart Assoc..

[12] Greenland P., Daviglus M.L., Dyer A.R., Liu K., Huang C.F., Goldberger J.J., Stamler J. (1999). Resting Heart Rate Is a Risk Factor for Cardiovascular and Noncardiovascular Mortality: The Chicago Heart Association Detection Project in Industry. Am. J. Epidemiol..

[13] Gutierrez-Martinez L., Brellenthin A.G., Lefferts E.C., Lee D., Sui X., Lavie C.J., Blair S.N. (2021). Resting Heart Rate and Risk of Cancer Mortality. Cancer Epidemiol. Biomark. Prev..

[14] Zhang D., Wang W., Li F. (2016). Association between Resting Heart Rate and Coronary Artery Disease, Stroke, Sudden Death and Noncardiovascular Diseases: A Meta-Analysis. CMAJ.

[15] Fox K., Borer J.S., Camm A.J., Danchin N., Ferrari R., Lopez Sendon J.L., Steg P.G., Tardif J.-C., Tavazzi L., Tendera M. (2007). Heart Rate Working Group. Resting Heart Rate in Cardiovascular Disease. J. Am. Coll. Cardiol..

[16] Seccareccia F., Pannozzo F., Dima F., Minoprio A., Menditto A., Lo Noce C., Giampaoli S. (2001). Malattie Cardiovascolari Aterosclerotiche Istituto Superiore di Sanita Project. Heart Rate as a Predictor of Mortality: The MATISS Project. Am. J. Public Health.

[17] Wod M., Jensen M.T., Galatius S., Hjelmborg J.B., Jensen G.B., Christensen K. (2019). Resting Heart Rate and Mortality in the Very Old. Scand. J. Clin. Lab. Investig..

[18] Ristow B., Doubell A., Derman W., Heine M. (2022). Change in Resting Heart Rate and Risk for All-Cause Mortality. Eur. J. Prev. Cardiol..

[19] Raisi-Estabragh Z., Cooper J., Judge R., Khanji M.Y., Munroe P.B., Cooper C., Harvey N.C., Petersen S.E. (2020). Age, Sex and Disease-Specific Associations between Resting Heart Rate and Cardiovascular Mortality in the UK BIOBANK. PLoS ONE.

[20] Puig E., Clará A., Pérez S., Degano I.R., Subirana I., García-García C., Martí R., Ramos R., Marrugat J., Elosua R. (2022). Resting Heart Rate, Cardiovascular Events, and All-Cause Mortality: The REGICOR Study. Eur. J. Prev. Cardiol..

[21] Okamura T., Hayakawa T., Kadowaki T., Kita Y., Okayama A., Elliott P., Ueshima H. (2004). Resting Heart Rate and Cause-Specific Death in a 16.5-Year Cohort Study of the Japanese General Population. Am. Heart J..

[22] Woodward M., Webster R., Murakami Y., Barzi F., Lam T.-H., Fang X., Suh I., Batty G.D., Huxley R., Rodgers A. (2014). The Association between Resting Heart Rate, Cardiovascular Disease and Mortality: Evidence from 112,680 Men and Women in 12 Cohorts. Eur. J. Prev. Cardiol..

[23] Vazir A., Claggett B., Cheng S., Skali H., Shah A., Agulair D., Ballantyne C.M., Vardeny O., Solomon S.D. (2018). Association of Resting Heart Rate and Temporal Changes in Heart Rate With Outcomes in Participants of the Atherosclerosis Risk in Communities Study. JAMA Cardiol..

[24] Jensen M.T., Marott J.L., Allin K.H., Nordestgaard B.G., Jensen G.B. (2012). Resting Heart Rate Is Associated with Cardiovascular and All-Cause Mortality after Adjusting for Inflammatory Markers: The Copenhagen City Heart Study. Eur. J. Prev. Cardiol..

[25] Tverdal A., Hjellvik V., Selmer R. (2008). Heart Rate and Mortality from Cardiovascular Causes: A 12 Year Follow-up Study of 379,843 Men and Women Aged 40-45 Years. Eur. Heart J..

[26] He K., Chen X., Shi Z., Shi S., Tian Q., Hu X., Song R., Bai K., Shi W., Wang J. (2022). Relationship of Resting Heart Rate and Blood Pressure with All-Cause and Cardiovascular Disease Mortality. Public Health.

[27] Hartaigh B.Ó., Gill T.M., Shah I., Hughes A.D., Deanfield J.E., Kuh D., Hardy R. (2014). Association between Resting Heart Rate across the Life Course and All-Cause Mortality: Longitudinal Findings from the Medical Research Council (MRC) National Survey of Health and Development (NSHD). J. Epidemiol. Community Health.

[28] Cui X., Mandalenakis Z., Thunström E., Fu M., Svärdsudd K., Hansson P.-O. (2021). The Impact of Time-Updated Resting Heart Rate on Cause-Specific Mortality in a Random Middle-Aged Male Population: A Lifetime Follow-Up. Clin. Res. Cardiol..

[29] Aune D., Sen A., ó’Hartaigh B., Janszky I., Romundstad P.R., Tonstad S., Vatten L.J. (2017). Resting Heart Rate and the Risk of Cardiovascular Disease, Total Cancer, and All-Cause Mortality—A Systematic Review and Dose–Response Meta-Analysis of Prospective Studies. Nutr. Metab. Cardiovasc. Dis..

[30] Kizilbash M.A., Daviglus M.L., Dyer A.R., Garside D.B., Hankinson A.L., Yan L.L., Tian L., Van L., Wang R., Greenland P. (2008). Relation of Heart Rate With Cardiovascular Disease in Normal-Weight Individuals: The Chicago Heart Association Detection Project in Industry. Prev. Cardiol..

[31] Custodis F., Reil J.-C., Laufs U., Böhm M. (2013). Heart Rate: A Global Target for Cardiovascular Disease and Therapy along the Cardiovascular Disease Continuum. J. Cardiol..

[32] Böhm M., Schumacher H., Teo K.K., Lonn E.M., Mahfoud F., Ukena C., Mann J.F.E., Mancia G., Redon J., Schmieder R.E. (2020). Resting Heart Rate and Cardiovascular Outcomes in Diabetic and Non-Diabetic Individuals at High Cardiovascular Risk Analysis from the ONTARGET/TRANSCEND Trials. Eur. Heart J..

[33] Floyd J.S., Sitlani C.M., Wiggins K.L., Wallace E., Suchy-Dicey A., Abbasi S.A., Carnethon M.R., Siscovick D.S., Sotoodehnia N., Heckbert S.R. (2015). Variation in Resting Heart Rate over Four Years and the Risks of Myocardial Infarction and Death among Older Adults. Heart.

[34] Samuel M., Arif S.G., Afilalo J. (2025). Heart Rate Variability as a Digital Biomarker for Frailty in Cardiovascular Patients. J. Frailty Aging.

[35] Yang X., Hidru T.H., Han X., Zhang X., Liu Y., Wang B., Li H., Wu S., Xia Y.-L. (2020). Link Between Elevated Long-Term Resting Heart Rate Variability and Pulse Pressure Variability for All-Cause Mortality. J. Am. Heart Assoc..

[36] Sacha J. (2014). Interaction between Heart Rate and Heart Rate Variability. Ann. Noninvasive Electrocardiol..

[37] Rocha A.S.L., Siqueira V.d.B., Maduro P.A., Batista L.d.S.P., Schwingel P.A. (2024). Reference Values for Heart Rate Variability in Older Adults: A Systematic Review. Psychophysiology.

[38] Nwabuo C.C., Appiah D., Moreira H.T., Vasconcellos H.D., Aghaji Q.N., Ambale-Venkatesh B., Rana J.S., Allen N.B., Lloyd-Jones D.M., Schreiner P.J. (2020). Temporal Changes in Resting Heart Rate, Left Ventricular Dysfunction, Heart Failure and Cardiovascular Disease: CARDIA Study. Am. J. Med..

[39] Míková M., Pospíšil D., Řehoř J., Malik M. (2025). Heart Rate Variability Analysis in Congestive Heart Failure: The Need for Standardized Assessment Protocols. Rev. Cardiovasc. Med..

[40] Koopman J.J.E., van Bodegom D., Maan A.C., Li Z., Ziem J.B., Westendorp R.G.J., Jukema J.W. (2015). Heart Rate Variability, but Not Heart Rate, Is Associated with Handgrip Strength and Mortality in Older Africans at Very Low Cardiovascular Risk: A Population-Based Study. Int. J. Cardiol..

[41] Jouven X., Empana J.P., Escolano S., Buyck J.F., Tafflet M., Desnos M., Ducimetière P. (2009). Relation of Heart Rate at Rest and Long-Term (>20 Years) Death Rate in Initially Healthy Middle-Aged Men. Am. J. Cardiol..

[42] Khan H., Kunutsor S., Kalogeropoulos A.P., Georgiopoulou V.V., Newman A.B., Harris T.B., Bibbins-Domingo K., Kauhanen J., Gheorghiade M., Fonarow G.C. (2015). Resting Heart Rate and Risk of Incident Heart Failure: Three Prospective Cohort Studies and a Systematic Meta-Analysis. J. Am. Heart Assoc..

[43] Jarczok M.N., Weimer K., Braun C., Williams D.P., Thayer J.F., Gündel H.O., Balint E.M. (2022). Heart Rate Variability in the Prediction of Mortality: A Systematic Review and Meta-Analysis of Healthy and Patient Populations. Neurosci. Biobehav. Rev..

[44] Pittaras A.M., Faselis C., Doumas M., Myers J., Kheirbek R., Kokkinos J.P., Tsimploulis A., Aiken M., Kokkinos P. (2013). Heart Rate at Rest, Exercise Capacity, and Mortality Risk in Veterans. Am. J. Cardiol..

[45] Saxena A., Minton D., Lee D., Sui X., Fayad R., Lavie C.J., Blair S.N. (2013). Protective Role of Resting Heart Rate on All-Cause and Cardiovascular Disease Mortality. Mayo Clin. Proc..

[46] Choi Y., Kim G., Yoon J., Kim Y.S. (2024). Association of Resting Heart Rate and Physical Activity with Cardiovascular Mortality: A Population-Based Cohort Study of Korean Adults. J. Sports Sci..

[47] Janssen I., Katzmarzyk P.T., Church T.S., Blair S.N. (2005). The Cooper Clinic Mortality Risk Index: Clinical Score Sheet for Men. Am. J. Prev. Med..

[48] Mancia G., Masi S., Palatini P., Tsioufis C., Grassi G. (2021). Elevated Heart Rate and Cardiovascular Risk in Hypertension. J. Hypertens..

[49] Grassi G., Ram C.V.S., Palatini P. (2025). High Heart Rate, Sympathetic Overdrive, and Cardiovascular Risk in Hypertension. Am. J. Cardiol..

[50] Osibogun O., Ogunmoroti O., Spatz E.S., Fashanu O.E., Michos E.D. (2020). Ideal Cardiovascular Health and Resting Heart Rate in the Multi-Ethnic Study of Atherosclerosis. Prev. Med..

[51] Lin C.-H., Zhang J.-F., Kuo Y.-W., Kuo C.-F., Huang Y.-C., Lee M., Lee J.-D. (2024). Assessment of the Impact of Resting Heart Rate on the Risk of Major Adverse Cardiovascular Events after Ischemic Stroke: A Retrospective Observational Study. BMC Neurol..

[52] Zambach C., Fedorowski A., Borné Y., Johnson L.S.B., Gerward S., Hamrefors V., Engström G. (2021). Cardiovascular Risk Factors and Autonomic Indices in Relation to Fatal and Non-Fatal Coronary Events. Open Heart.

[53] Memenga F., Rybczynski M., Magnussen C., Goßling A., Kondziella C., Becher N., Becher P.M., Bernadyn J., Berisha F., Bremer W. (2023). Heart Rate Reduction and Outcomes in Heart Failure Outpatients. J. Clin. Med..

[54] Saito H., Tanaka K., Ejiri H., Kimura H., Shimabukuro M., Asahi K., Watanabe T., Kazama J.J. (2024). Elevated Resting Heart Rate Is Associated with Mortality in Patients with Chronic Kidney Disease. Sci. Rep..

[55] Omlor A.J., Trudzinski F.C., Alqudrah M., Seiler F., Biertz F., Vogelmeier C.F., Welte T., Watz H., Waschki B., Brinker T.J. (2020). Time-Updated Resting Heart Rate Predicts Mortality in Patients with COPD. Clin. Res. Cardiol..

[56] McEvoy J.W., McCarthy C.P., Bruno R.M., Brouwers S., Canavan M.D., Ceconi C., Christodorescu R.M., Daskalopoulou S.S., Ferro C.J., Gerdts E. (2024). 2024 ESC Guidelines for the Management of Elevated Blood Pressure and Hypertension. Eur. Heart J..

[57] Palazón-Bru A., Ferri-Rufete D., Mares-García E., Durazo-Arvizu R.Á., Divisón-Garrote J.A., Carbayo-Herencia J.A., Artigao-Rodenas L.M., Simarro-Rueda M., Molina-Escribano F., Ponce-García I. (2020). Clusters of Cardiovascular Risk Factors and Their Impact on the 20-Year Cardiovascular Risk in a General Population. J. Cardiovasc. Nurs..

[58] Artigao-Ródenas L.M., Carbayo-Herencia J.A., Palazón-Bru A., Divisón-Garrote J.A., Sanchis-Domènech C., Vigo-Aguiar I., Gil-Guillén V.F. (2015). Construction and Validation of a 14-Year Cardiovascular Risk Score for Use in the General Population: The Puras-GEVA Chart. Medicine.

[59] Akbarpour S., Azizpour Y., Larijani B. (2025). Global Effect of Cardiovascular Risk Factors on Lifetime Estimates. N. Engl. J. Med..

[60] Kleinbaum D. (2005). Evaluating the Proportional Hazards Assumption. Survival Analysis: A Self-Learning Text.

[61] Shigetoh Y., Adachi H., Yamagishi S., Enomoto M., Fukami A., Otsuka M., Kumagae S., Furuki K., Nanjo Y., Imaizumi T. (2009). Higher Heart Rate May Predispose to Obesity and Diabetes Mellitus: 20-Year Prospective Study in a General Population. Am. J. Hypertens..

[62] Palatini P. (2025). High Heart Rate and Hypertension: Two Intertwined Cardiovascular Risk Factors. J. Clin. Hypertens..

[63] Wu X., Du R., Hu C., Cheng D., Ma L., Li M., Xu Y., Xu M., Chen Y., Li D. (2019). Resting Heart Rate Is Associated with Metabolic Syndrome and Predicted 10-Year Risk of Cardiovascular Disease: A Cross-Sectional Study. J. Diabetes.

[64] Zamorano J.L. (2008). Heart Rate Management: A Therapeutic Goal throughout the Cardiovascular Continuum. Eur. Heart J. Suppl..

[65] Böhm M., Reil J.-C., Deedwania P., Kim J.B., Borer J.S. (2015). Resting Heart Rate: Risk Indicator and Emerging Risk Factor in Cardiovascular Disease. Am. J. Med..

[66] Olshansky B., Ricci F., Fedorowski A. (2023). Importance of Resting Heart Rate. Trends Cardiovasc. Med..

